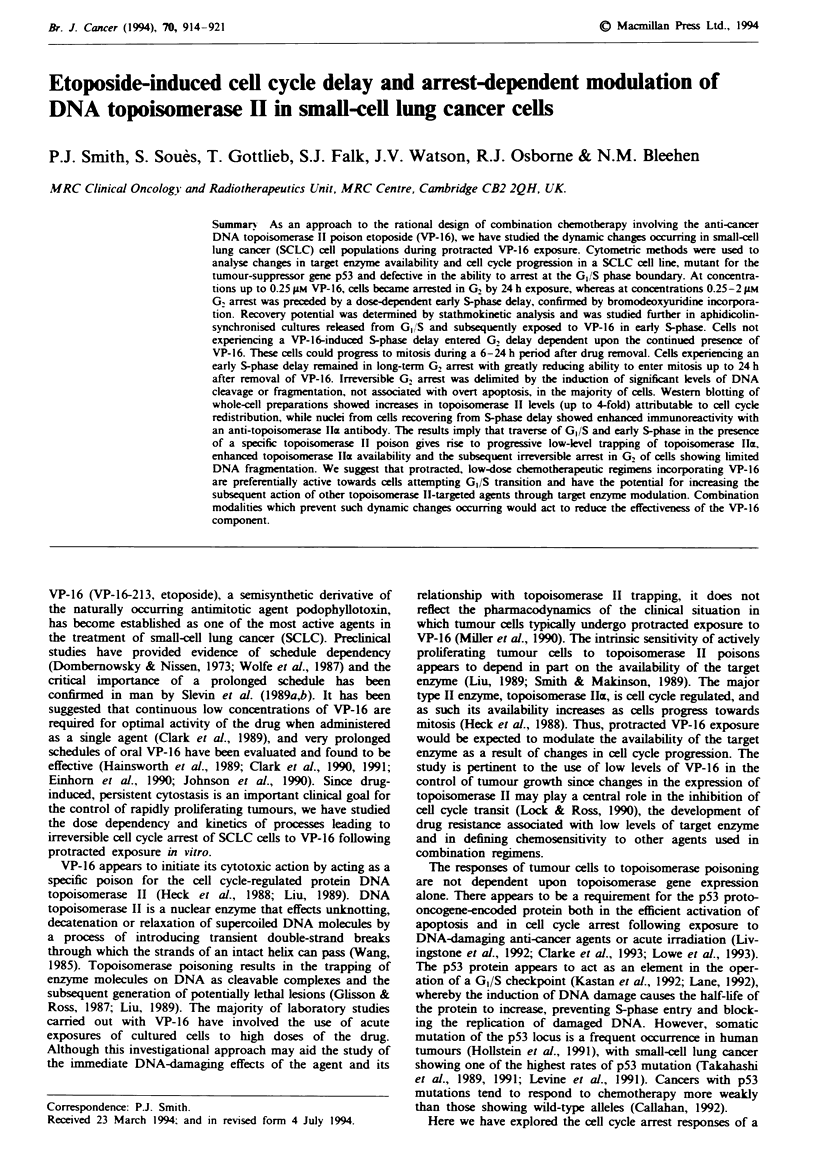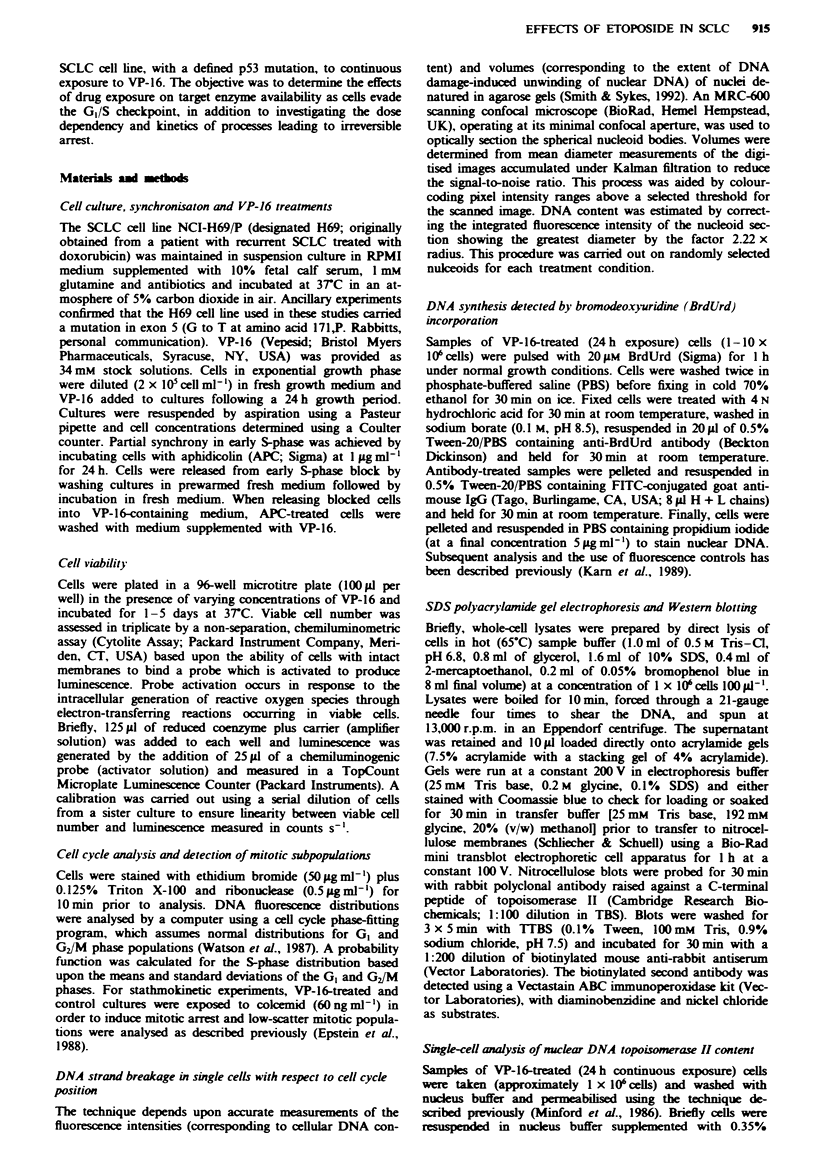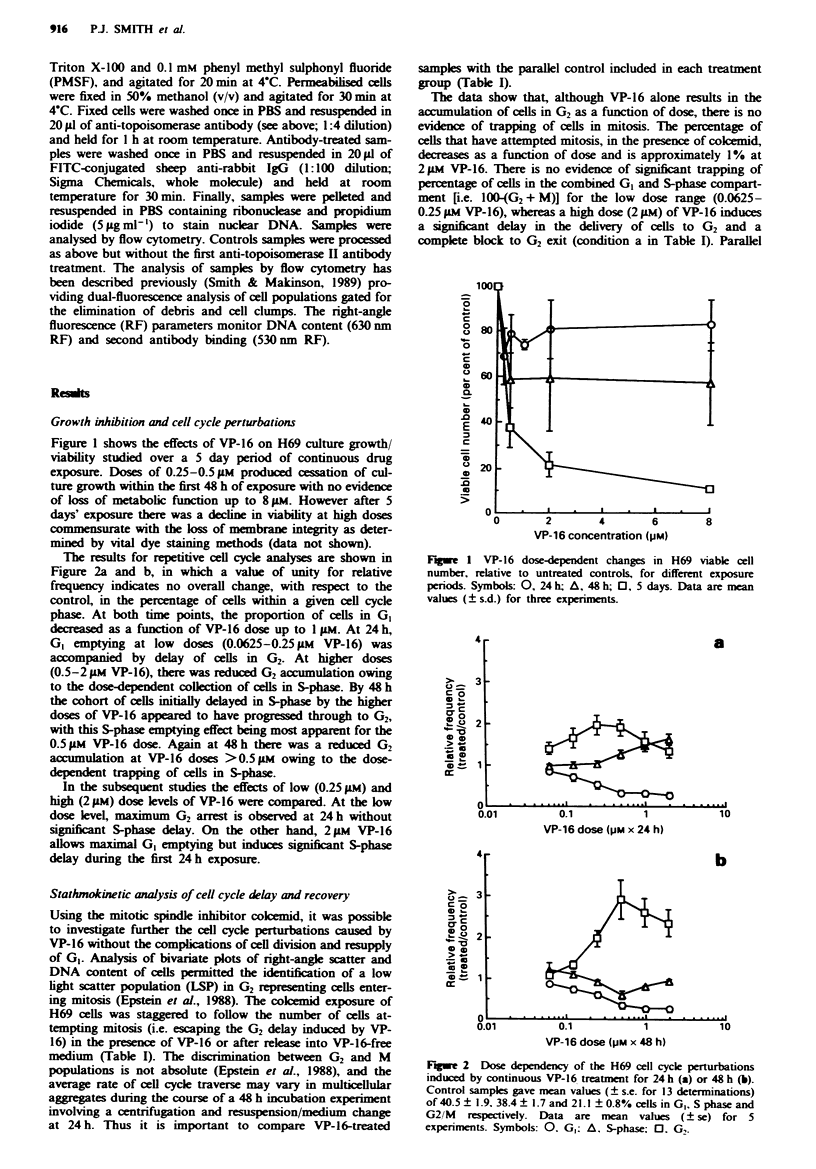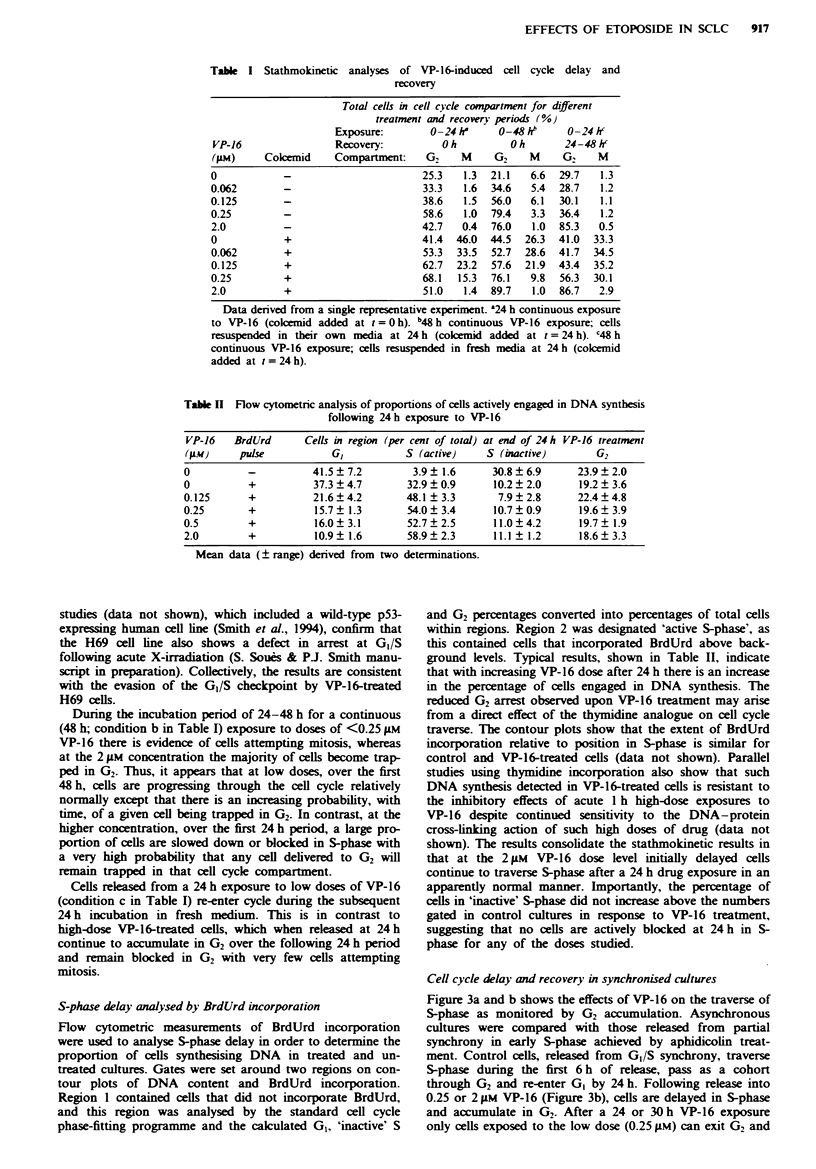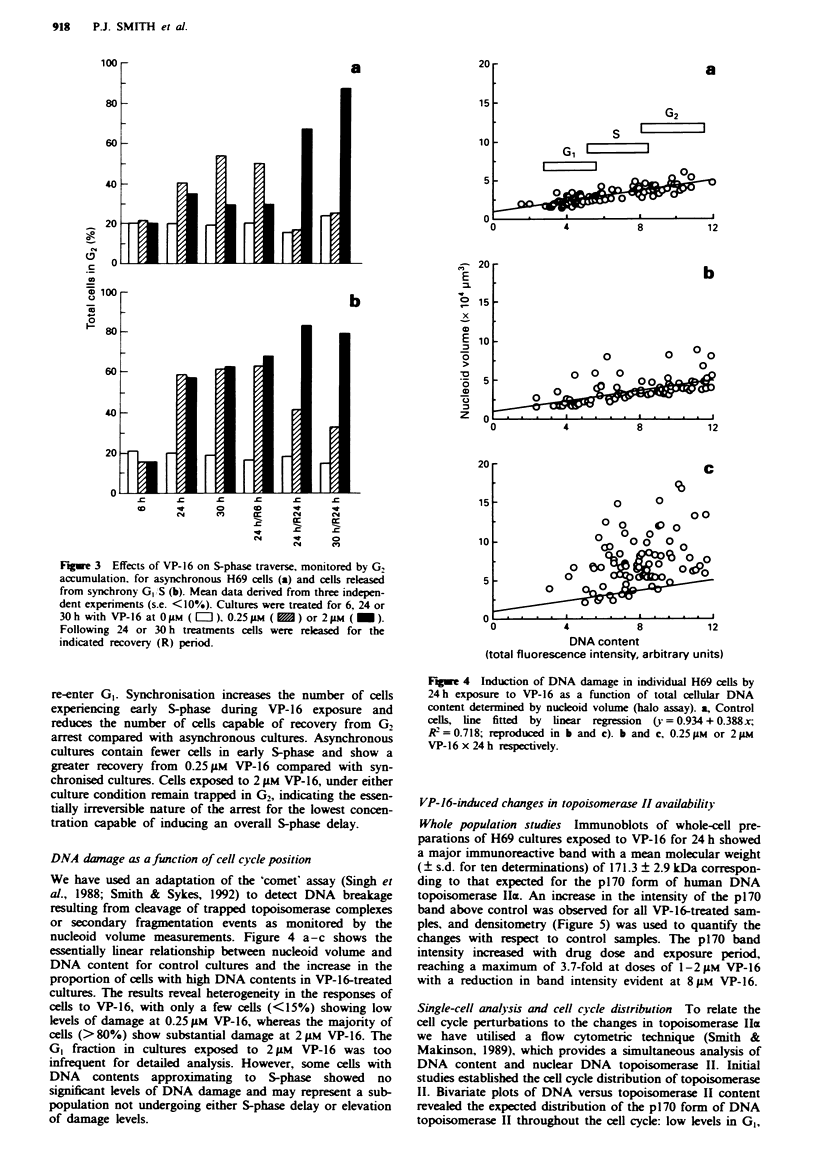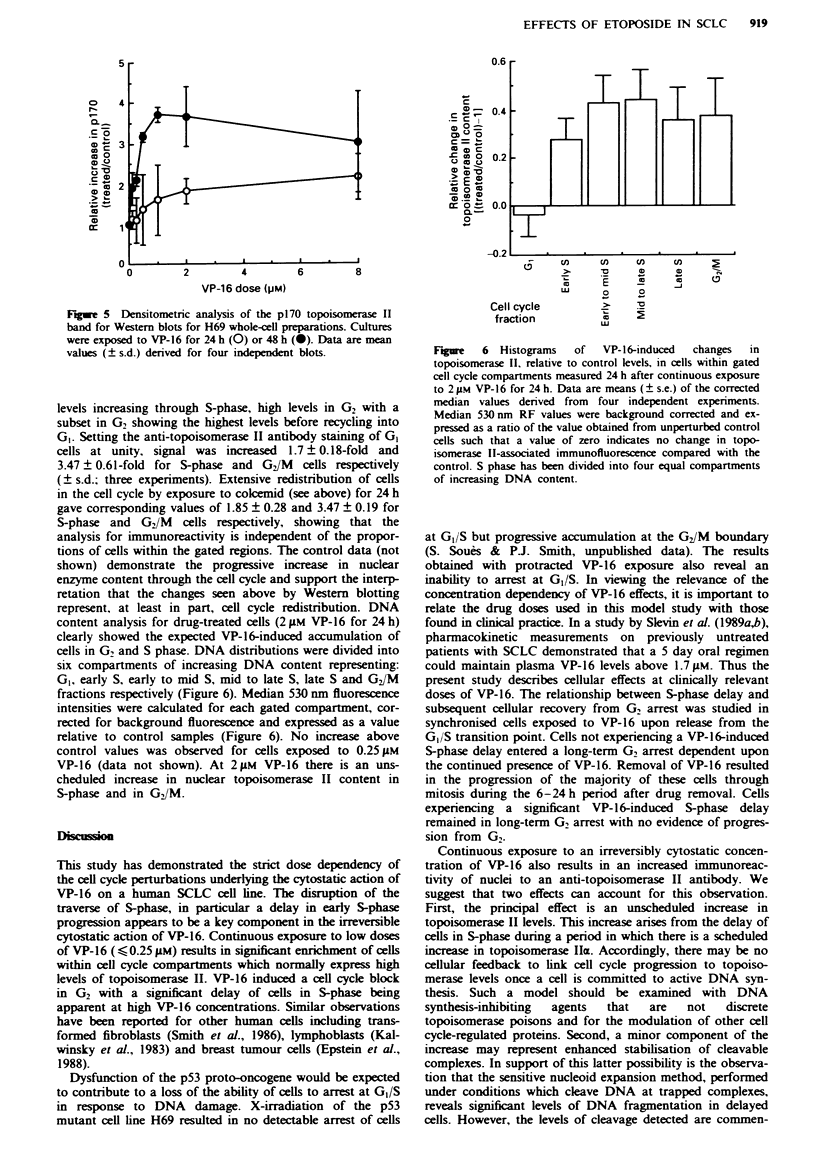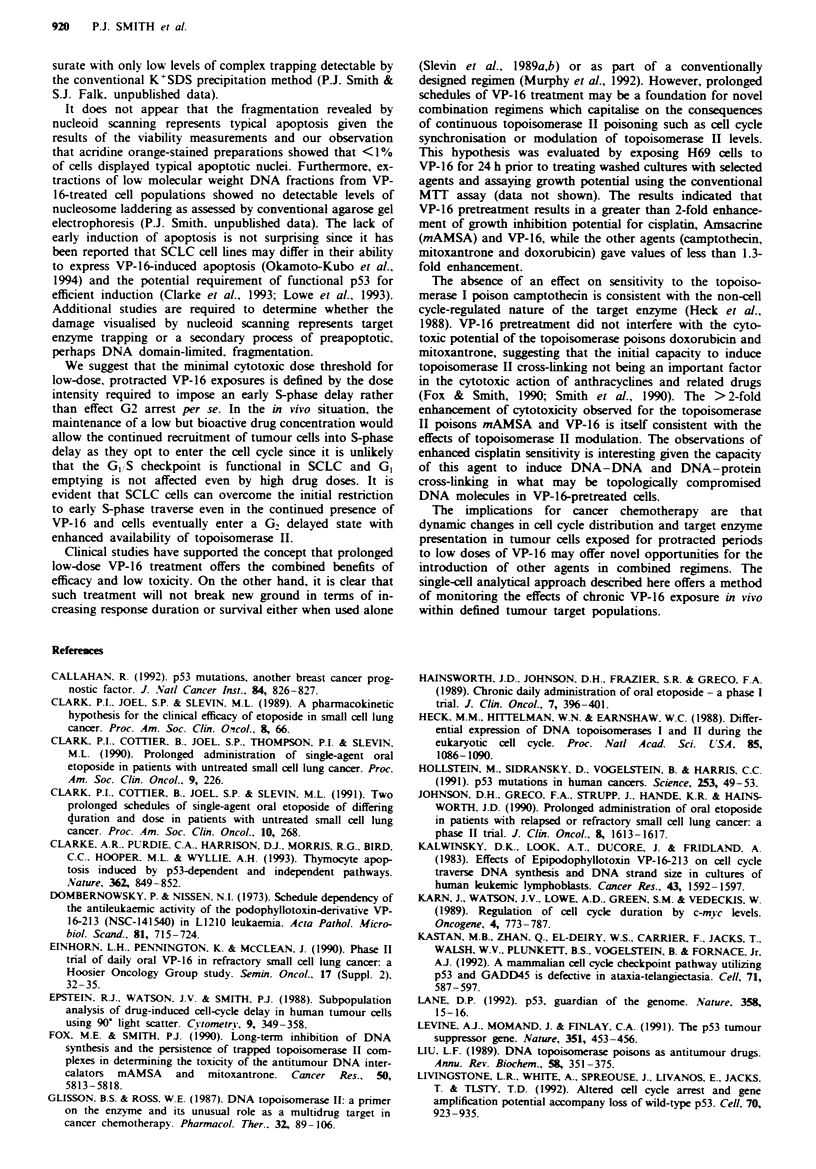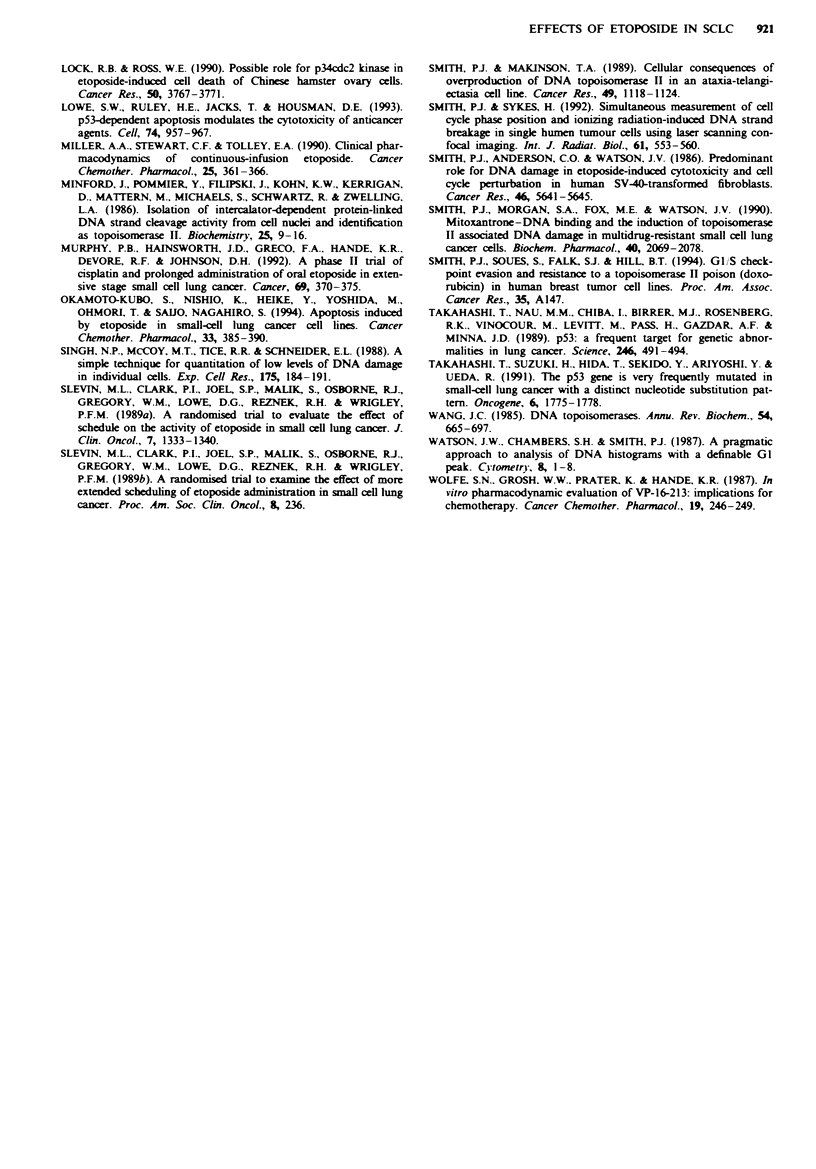# Etoposide-induced cell cycle delay and arrest-dependent modulation of DNA topoisomerase II in small-cell lung cancer cells.

**DOI:** 10.1038/bjc.1994.420

**Published:** 1994-11

**Authors:** P. J. Smith, S. Souès, T. Gottlieb, S. J. Falk, J. V. Watson, R. J. Osborne, N. M. Bleehen

**Affiliations:** MRC Clinical Oncology and Radiotherapeutics Unit, MRC Centre, Cambridge, UK.

## Abstract

As an approach to the rational design of combination chemotherapy involving the anti-cancer DNA topoisomerase II poison etoposide (VP-16), we have studied the dynamic changes occurring in small-cell lung cancer (SCLC) cell populations during protracted VP-16 exposure. Cytometric methods were used to analyse changes in target enzyme availability and cell cycle progression in a SCLC cell line, mutant for the tumour-suppressor gene p53 and defective in the ability to arrest at the G1/S phase boundary. At concentrations up to 0.25 microM VP-16, cells became arrested in G2 by 24 h exposure, whereas at concentrations 0.25-2 microM G2 arrest was preceded by a dose-dependent early S-phase delay, confirmed by bromodeoxyuridine incorporation. Recovery potential was determined by stathmokinetic analysis and was studied further in aphidicolin-synchronised cultures released from G1/S and subsequently exposed to VP-16 in early S-phase. Cells not experiencing a VP-16-induced S-phase delay entered G2 delay dependent upon the continued presence of VP-16. These cells could progress to mitosis during a 6-24 h period after drug removal. Cells experiencing an early S-phase delay remained in long-term G2 arrest with greatly reducing ability to enter mitosis up to 24 h after removal of VP-16. Irreversible G2 arrest was delimited by the induction of significant levels of DNA cleavage or fragmentation, not associated with overt apoptosis, in the majority of cells. Western blotting of whole-cell preparations showed increases in topoisomerase II levels (up to 4-fold) attributable to cell cycle redistribution, while nuclei from cells recovering from S-phase delay showed enhanced immunoreactivity with an anti-topoisomerase II alpha antibody. The results imply that traverse of G1/S and early S-phase in the presence of a specific topoisomerase II poison gives rise to progressive low-level trapping of topoisomerase II alpha, enhanced topoisomerase II alpha availability and the subsequent irreversible arrest in G2 of cells showing limited DNA fragmentation. We suggest that protracted, low-dose chemotherapeutic regimens incorporating VP-16 are preferentially active towards cells attempting G1/S transition and have the potential for increasing the subsequent action of other topoisomerase II-targeted agents through target enzyme modulation. Combination modalities which prevent such dynamic changes occurring would act to reduce the effectiveness of the VP-16 component.


© Macmillan Press Ltd., 1994

### Etoposide-induced cell cycle delay and arrest-dependent modulation of DNA topoisomerase II in small-cell lung cancer cells

P.J. Smith, S. Souès, T. Gottlieb, S.J. Falk, J.V. Watson, R.J. Osborne & N.M. Bleehen

*MRC Clinical Oncology and Radiotherapeutics Unit, MRC Centre, Cambridge CB2 2QH, UK.*

**Summary** As an approach to the rational design of combination chemotherapy involving the anti-cancer DNA topoisomerase II poison etoposide (VP-16), we have studied the dynamic changes occurring in small-cell lung cancer (SCLC) cell populations during protracted VP-16 exposure. Cytometric methods were used to analyse changes in target enzyme availability and cell cycle progression in a SCLC cell line, mutant for the tumour-suppressor gene p53 and defective in the ability to arrest at the $G_1/S$ phase boundary. At concentrations up to 0.25 μM VP-16, cells became arrested in $G_2$ by 24 h exposure, whereas at concentrations 0.25–2 μM $G_2$ arrest was preceded by a dose-dependent early S-phase delay, confirmed by bromodeoxyuridine incorporation. Recovery potential was determined by stathmokinetic analysis and was studied further in aphidicolin-synchronised cultures released from $G_1/S$ and subsequently exposed to VP-16 in early S-phase. Cells not experiencing a VP-16-induced S-phase delay entered $G_2$ delay dependent upon the continued presence of VP-16. These cells could progress to mitosis during a 6–24 h period after drug removal. Cells experiencing an early S-phase delay remained in long-term $G_2$ arrest with greatly reducing ability to enter mitosis up to 24 h after removal of VP-16. Irreversible $G_2$ arrest was delimited by the induction of significant levels of DNA cleavage or fragmentation, not associated with overt apoptosis, in the majority of cells. Western blotting of whole-cell preparations showed increases in topoisomerase II levels (up to 4-fold) attributable to cell cycle redistribution, while nuclei from cells recovering from S-phase delay showed enhanced immunoreactivity with an anti-topoisomerase IIα antibody. The results imply that traverse of $G_1/S$ and early S-phase in the presence of a specific topoisomerase II poison gives rise to progressive low-level trapping of topoisomerase IIα, enhanced topoisomerase IIα availability and the subsequent irreversible arrest in $G_2$ of cells showing limited DNA fragmentation. We suggest that protracted, low-dose chemotherapeutic regimens incorporating VP-16 are preferentially active towards cells attempting $G_1/S$ transition and have the potential for increasing the subsequent action of other topoisomerase II-targeted agents through target enzyme modulation. Combination modalities which prevent such dynamic changes occurring would act to reduce the effectiveness of the VP-16 component.

VP-16 (VP-16-213, etoposide), a semisynthetic derivative of the naturally occurring antimitotic agent podophyllotoxin, has become established as one of the most active agents in the treatment of small-cell lung cancer (SCLC). Preclinical studies have provided evidence of schedule dependency (Dombernowsky & Nissen, 1973; Wolfe *et al.*, 1987) and the critical importance of a prolonged schedule has been confirmed in man by Slevin *et al.* (1989*a,b*). It has been suggested that continuous low concentrations of VP-16 are required for optimal activity of the drug when administered as a single agent (Clark *et al.*, 1989), and very prolonged schedules of oral VP-16 have been evaluated and found to be effective (Hainsworth *et al.*, 1989; Clark *et al.*, 1990, 1991; Einhorn *et al.*, 1990; Johnson *et al.*, 1990). Since drug-induced, persistent cytostasis is an important clinical goal for the control of rapidly proliferating tumours, we have studied the dose dependency and kinetics of processes leading to irreversible cell cycle arrest of SCLC cells to VP-16 following protracted exposure *in vitro*.

VP-16 appears to initiate its cytotoxic action by acting as a specific poison for the cell cycle-regulated protein DNA topoisomerase II (Heck *et al.*, 1988; Liu, 1989). DNA topoisomerase II is a nuclear enzyme that effects unknotting, decatenation or relaxation of supercoiled DNA molecules by a process of introducing transient double-strand breaks through which the strands of an intact helix can pass (Wang, 1985). Topoisomerase poisoning results in the trapping of enzyme molecules on DNA as cleavable complexes and the subsequent generation of potentially lethal lesions (Glisson & Ross, 1987; Liu, 1989). The majority of laboratory studies carried out with VP-16 have involved the use of acute exposures of cultured cells to high doses of the drug. Although this investigational approach may aid the study of the immediate DNA-damaging effects of the agent and its relationship with topoisomerase II trapping, it does not reflect the pharmacodynamics of the clinical situation in which tumour cells typically undergo protracted exposure to VP-16 (Miller *et al.*, 1990). The intrinsic sensitivity of actively proliferating tumour cells to topoisomerase II poisons appears to depend in part on the availability of the target enzyme (Liu, 1989; Smith & Makinson, 1989). The major type II enzyme, topoisomerase IIα, is cell cycle regulated, and as such its availability increases as cells progress towards mitosis (Heck *et al.*, 1988). Thus, protracted VP-16 exposure would be expected to modulate the availability of the target enzyme as a result of changes in cell cycle progression. The study is pertinent to the use of low levels of VP-16 in the control of tumour growth since changes in the expression of topoisomerase II may play a central role in the inhibition of cell cycle transit (Lock & Ross, 1990), the development of drug resistance associated with low levels of target enzyme and in defining chemosensitivity to other agents used in combination regimens.

The responses of tumour cells to topoisomerase poisoning are not dependent upon topoisomerase gene expression alone. There appears to be a requirement for the p53 proto-oncogene-encoded protein both in the efficient activation of apoptosis and in cell cycle arrest following exposure to DNA-damaging anti-cancer agents or acute irradiation (Livingstone *et al.*, 1992; Clarke *et al.*, 1993; Lowe *et al.*, 1993). The p53 protein appears to act as an element in the operation of a $G_1/S$ checkpoint (Kastan *et al.*, 1992; Lane, 1992), whereby the induction of DNA damage causes the half-life of the protein to increase, preventing S-phase entry and blocking the replication of damaged DNA. However, somatic mutation of the p53 locus is a frequent occurrence in human tumours (Hollstein *et al.*, 1991), with small-cell lung cancer showing one of the highest rates of p53 mutation (Takahashi *et al.*, 1989, 1991; Levine *et al.*, 1991). Cancers with p53 mutations tend to respond to chemotherapy more weakly than those showing wild-type alleles (Callahan, 1992).

Here we have explored the cell cycle arrest responses of a

Correspondence: P.J. Smith.
Received 23 March 1994; and in revised form 4 July 1994.

SCLC cell line, with a defined p53 mutation, to continuous exposure to VP-16. The objective was to determine the effects of drug exposure on target enzyme availability as cells evade the $G_1/S$ checkpoint, in addition to investigating the dose dependency and kinetics of processes leading to irreversible arrest.

### Materials and methods

### *Cell culture, synchronisaton and VP-16 treatments*

The SCLC cell line NCI-H69/P (designated H69; originally obtained from a patient with recurrent SCLC treated with doxorubicin) was maintained in suspension culture in RPMI medium supplemented with 10% fetal calf serum, 1 mM glutamine and antibiotics and incubated at 37°C in an atmosphere of 5% carbon dioxide in air. Ancillary experiments confirmed that the H69 cell line used in these studies carried a mutation in exon 5 (G to T at amino acid 171,P. Rabbitts, personal communication). VP-16 (Vepesid; Bristol Myers Pharmaceuticals, Syracuse, NY, USA) was provided as 34 mM stock solutions. Cells in exponential growth phase were diluted ($2 \times 10^5$ cell ml$^{-1}$) in fresh growth medium and VP-16 added to cultures following a 24 h growth period. Cultures were resuspended by aspiration using a Pasteur pipette and cell concentrations determined using a Coulter counter. Partial synchrony in early S-phase was achieved by incubating cells with aphidicolin (APC; Sigma) at 1 $\mu$g ml$^{-1}$ for 24 h. Cells were released from early S-phase block by washing cultures in prewarmed fresh medium followed by incubation in fresh medium. When releasing blocked cells into VP-16-containing medium, APC-treated cells were washed with medium supplemented with VP-16.

### *Cell viability*

Cells were plated in a 96-well microtitre plate (100 $\mu$l per well) in the presence of varying concentrations of VP-16 and incubated for 1–5 days at 37°C. Viable cell number was assessed in triplicate by a non-separation, chemiluminometric assay (Cytolite Assay; Packard Instrument Company, Meriden, CT, USA) based upon the ability of cells with intact membranes to bind a probe which is activated to produce luminescence. Probe activation occurs in response to the intracellular generation of reactive oxygen species through electron-transferring reactions occurring in viable cells. Briefly, 125 $\mu$l of reduced coenzyme plus carrier (amplifier solution) was added to each well and luminescence was generated by the addition of 25 $\mu$l of a chemiluminogenic probe (activator solution) and measured in a TopCount Microplate Luminescence Counter (Packard Instruments). A calibration was carried out using a serial dilution of cells from a sister culture to ensure linearity between viable cell number and luminescence measured in counts s$^{-1}$.

### *Cell cycle analysis and detection of mitotic subpopulations*

Cells were stained with ethidium bromide (50 $\mu$g ml$^{-1}$) plus 0.125% Triton X-100 and ribonuclease (0.5 $\mu$g ml$^{-1}$) for 10 min prior to analysis. DNA fluorescence distributions were analysed by a computer using a cell cycle phase-fitting program, which assumes normal distributions for $G_1$ and $G_2/M$ phase populations (Watson *et al.*, 1987). A probability function was calculated for the S-phase distribution based upon the means and standard deviations of the $G_1$ and $G_2/M$ phases. For stathmokinetic experiments, VP-16-treated and control cultures were exposed to colcemid (60 ng ml$^{-1}$) in order to induce mitotic arrest and low-scatter mitotic populations were analysed as described previously (Epstein *et al.*, 1988).

### *DNA strand breakage in single cells with respect to cell cycle position*

The technique depends upon accurate measurements of the fluorescence intensities (corresponding to cellular DNA content) and volumes (corresponding to the extent of DNA damage-induced unwinding of nuclear DNA) of nuclei denatured in agarose gels (Smith & Sykes, 1992). An MRC-600 scanning confocal microscope (BioRad, Hemel Hempstead, UK), operating at its minimal confocal aperture, was used to optically section the spherical nucleoid bodies. Volumes were determined from mean diameter measurements of the digitised images accumulated under Kalman filtration to reduce the signal-to-noise ratio. This process was aided by colour-coding pixel intensity ranges above a selected threshold for the scanned image. DNA content was estimated by correcting the integrated fluorescence intensity of the nucleoid section showing the greatest diameter by the factor 2.22 × radius. This procedure was carried out on randomly selected nulkeoids for each treatment condition.

### *DNA synthesis detected by bromodeoxyuridine (BrdUrd) incorporation*

Samples of VP-16-treated (24 h exposure) cells ($1-10 \times 10^6$ cells) were pulsed with 20 $\mu$M BrdUrd (Sigma) for 1 h under normal growth conditions. Cells were washed twice in phosphate-buffered saline (PBS) before fixing in cold 70% ethanol for 30 min on ice. Fixed cells were treated with 4 N hydrochloric acid for 30 min at room temperature, washed in sodium borate (0.1 M, pH 8.5), resuspended in 20 $\mu$l of 0.5% Tween-20/PBS containing anti-BrdUrd antibody (Beckton Dickinson) and held for 30 min at room temperature. Antibody-treated samples were pelleted and resuspended in 0.5% Tween-20/PBS containing FITC-conjugated goat anti-mouse IgG (Tago, Burlingame, CA, USA; 8 $\mu$l H + L chains) and held for 30 min at room temperature. Finally, cells were pelleted and resuspended in PBS containing propidium iodide (at a final concentration 5 $\mu$g ml$^{-1}$) to stain nuclear DNA. Subsequent analysis and the use of fluorescence controls has been described previously (Karn *et al.*, 1989).

### *SDS polyacrylamide gel electrophoresis and Western blotting*

Briefly, whole-cell lysates were prepared by direct lysis of cells in hot (65°C) sample buffer (1.0 ml of 0.5 M Tris–Cl, pH 6.8, 0.8 ml of glycerol, 1.6 ml of 10% SDS, 0.4 ml of 2-mercaptoethanol, 0.2 ml of 0.05% bromophenol blue in 8 ml final volume) at a concentration of $1 \times 10^6$ cells 100 $\mu$l$^{-1}$. Lysates were boiled for 10 min, forced through a 21-gauge needle four times to shear the DNA, and spun at 13,000 r.p.m. in an Eppendorf centrifuge. The supernatant was retained and 10 $\mu$l loaded directly onto acrylamide gels (7.5% acrylamide with a stacking gel of 4% acrylamide). Gels were run at a constant 200 V in electrophoresis buffer (25 mM Tris base, 0.2 M glycine, 0.1% SDS) and either stained with Coomassie blue to check for loading or soaked for 30 min in transfer buffer [25 mM Tris base, 192 mM glycine, 20% (v/w) methanol] prior to transfer to nitrocellulose membranes (Schliecher & Schuell) using a Bio-Rad mini transblot electrophoretic cell apparatus for 1 h at a constant 100 V. Nitrocellulose blots were probed for 30 min with rabbit polyclonal antibody raised against a C-terminal peptide of topoisomerase II (Cambridge Research Biochemicals; 1:100 dilution in TBS). Blots were washed for 3 × 5 min with TTBS (0.1% Tween, 100 mM Tris, 0.9% sodium chloride, pH 7.5) and incubated for 30 min with a 1:200 dilution of biotinylated mouse anti-rabbit antiserum (Vector Laboratories). The biotinylated second antibody was detected using a Vectastain ABC immunoperoxidase kit (Vector Laboratories), with diaminobenzidine and nickel chloride as substrates.

### *Single-cell analysis of nuclear DNA topoisomerase II content*

Samples of VP-16-treated (24 h continuous exposure) cells were taken (approximately $1 \times 10^6$ cells) and washed with nucleus buffer and permeabilised using the technique described previously (Minford *et al.*, 1986). Briefly cells were resuspended in nucleus buffer supplemented with 0.35%

Triton X-100 and 0.1 mM phenyl methyl sulphonyl fluoride (PMSF), and agitated for 20 min at 4°C. Permeabilised cells were fixed in 50% methanol (v/v) and agitated for 30 min at 4°C. Fixed cells were washed once in PBS and resuspended in 20 µl of anti-topoisomerase antibody (see above; 1:4 dilution) and held for 1 h at room temperature. Antibody-treated samples were washed once in PBS and resuspended in 20 µl of FITC-conjugated sheep anti-rabbit IgG (1:100 dilution; Sigma Chemicals, whole molecule) and held at room temperature for 30 min. Finally, samples were pelleted and resuspended in PBS containing ribonuclease and propidium iodide ($5\,\mu g\,ml^{-1}$) to stain nuclear DNA. Samples were analysed by flow cytometry. Controls samples were processed as above but without the first anti-topoisomerase II antibody treatment. The analysis of samples by flow cytometry has been described previously (Smith & Makinson, 1989) providing dual-fluorescence analysis of cell populations gated for the elimination of debris and cell clumps. The right-angle fluorescence (RF) parameters monitor DNA content (630 nm RF) and second antibody binding (530 nm RF).

### Results

### *Growth inhibition and cell cycle perturbations*

Figure 1 shows the effects of VP-16 on H69 culture growth/viability studied over a 5 day period of continuous drug exposure. Doses of 0.25–0.5 µM produced cessation of culture growth within the first 48 h of exposure with no evidence of loss of metabolic function up to 8 µM. However after 5 days' exposure there was a decline in viability at high doses commensurate with the loss of membrane integrity as determined by vital dye staining methods (data not shown).

The results for repetitive cell cycle analyses are shown in Figure 2a and b, in which a value of unity for relative frequency indicates no overall change, with respect to the control, in the percentage of cells within a given cell cycle phase. At both time points, the proportion of cells in $G_1$ decreased as a function of VP-16 dose up to 1 µM. At 24 h, $G_1$ emptying at low doses (0.0625–0.25 µM VP-16) was accompanied by delay of cells in $G_2$. At higher doses (0.5–2 µM VP-16), there was reduced $G_2$ accumulation owing to the dose-dependent collection of cells in S-phase. By 48 h the cohort of cells initially delayed in S-phase by the higher doses of VP-16 appeared to have progressed through to $G_2$, with this S-phase emptying effect being most apparent for the 0.5 µM VP-16 dose. Again at 48 h there was a reduced $G_2$ accumulation at VP-16 doses $>0.5$ µM owing to the dose-dependent trapping of cells in S-phase.

In the subsequent studies the effects of low (0.25 µM) and high (2 µM) dose levels of VP-16 were compared. At the low dose level, maximum $G_2$ arrest is observed at 24 h without significant S-phase delay. On the other hand, 2 µM VP-16 allows maximal $G_1$ emptying but induces significant S-phase delay during the first 24 h exposure.

### *Stathmokinetic analysis of cell cycle delay and recovery*

Using the mitotic spindle inhibitor colcemid, it was possible to investigate further the cell cycle perturbations caused by VP-16 without the complications of cell division and resupply of $G_1$. Analysis of bivariate plots of right-angle scatter and DNA content of cells permitted the identification of a low light scatter population (LSP) in $G_2$ representing cells entering mitosis (Epstein et al., 1988). The colcemid exposure of H69 cells was staggered to follow the number of cells attempting mitosis (i.e. escaping the $G_2$ delay induced by VP-16) in the presence of VP-16 or after release into VP-16-free medium (Table I). The discrimination between $G_2$ and M populations is not absolute (Epstein et al., 1988), and the average rate of cell cycle traverse may vary in multicellular aggregates during the course of a 48 h incubation experiment involving a centrifugation and resuspension/medium change at 24 h. Thus it is important to compare VP-16-treated samples with the parallel control included in each treatment group (Table I).

The data show that, although VP-16 alone results in the accumulation of cells in $G_2$ as a function of dose, there is no evidence of trapping of cells in mitosis. The percentage of cells that have attempted mitosis, in the presence of colcemid, decreases as a function of dose and is approximately 1% at 2 µM VP-16. There is no evidence of significant trapping of percentage of cells in the combined $G_1$ and S-phase compartment [i.e. $100-(G_2 + M)$] for the low dose range (0.0625–0.25 µM VP-16), whereas a high dose (2 µM) of VP-16 induces a significant delay in the delivery of cells to $G_2$ and a complete block to $G_2$ exit (condition a in Table I). Parallel

**Figure 1** VP-16 dose-dependent changes in H69 viable cell number, relative to untreated controls, for different exposure periods. Symbols: ○, 24 h; △, 48 h; □, 5 days. Data are mean values (± s.d.) for three experiments.

**Figure 2** Dose dependency of the H69 cell cycle perturbations induced by continuous VP-16 treatment for 24 h (**a**) or 48 h (**b**). Control samples gave mean values (± s.e. for 13 determinations) of 40.5 ± 1.9, 38.4 ± 1.7 and 21.1 ± 0.8% cells in $G_1$, S phase and G2/M respectively. Data are mean values (± se) for 5 experiments. Symbols: ○, $G_1$; △, S-phase; □, $G_2$.

**Table I** Stathmokinetic analyses of VP-16-induced cell cycle delay and recovery

| VP-16 (μM) | Colcemid | | Exposure: 0–24 h[a] Recovery: 0 h $G_2$ | M | Exposure: 0–48 h[b] Recovery: 0 h $G_2$ | M | Exposure: 0–24 h[c] Recovery: 24–48 h[c] $G_2$ | M |
|---|---|---|---|---|---|---|---|---|
| | | *Total cells in cell cycle compartment for different treatment and recovery periods (%)* Compartment: | | | | | | |
| 0 | – | | 25.3 | 1.3 | 21.1 | 6.6 | 29.7 | 1.3 |
| 0.062 | – | | 33.3 | 1.6 | 34.6 | 5.4 | 28.7 | 1.2 |
| 0.125 | – | | 38.6 | 1.5 | 56.0 | 6.1 | 30.1 | 1.1 |
| 0.25 | – | | 58.6 | 1.0 | 79.4 | 3.3 | 36.4 | 1.2 |
| 2.0 | – | | 42.7 | 0.4 | 76.0 | 1.0 | 85.3 | 0.5 |
| 0 | + | | 41.4 | 46.0 | 44.5 | 26.3 | 41.0 | 33.3 |
| 0.062 | + | | 53.3 | 33.5 | 52.7 | 28.6 | 41.7 | 34.5 |
| 0.125 | + | | 62.7 | 23.2 | 57.6 | 21.9 | 43.4 | 35.2 |
| 0.25 | + | | 68.1 | 15.3 | 76.1 | 9.8 | 56.3 | 30.1 |
| 2.0 | + | | 51.0 | 1.4 | 89.7 | 1.0 | 86.7 | 2.9 |

Data derived from a single representative experiment. [a]24 h continuous exposure to VP-16 (colcemid added at $t = 0$ h). [b]48 h continuous VP-16 exposure; cells resuspended in their own media at 24 h (colcemid added at $t = 24$ h). [c]48 h continuous VP-16 exposure; cells resuspended in fresh media at 24 h (colcemid added at $t = 24$ h).

**Table II** Flow cytometric analysis of proportions of cells actively engaged in DNA synthesis following 24 h exposure to VP-16

| VP-16 (μM) | BrdUrd pulse | Cells in region (per cent of total) at end of 24 h VP-16 treatment: $G_1$ | S (active) | S (inactive) | $G_2$ |
|---|---|---|---|---|---|
| 0 | – | 41.5 ± 7.2 | 3.9 ± 1.6 | 30.8 ± 6.9 | 23.9 ± 2.0 |
| 0 | + | 37.3 ± 4.7 | 32.9 ± 0.9 | 10.2 ± 2.0 | 19.2 ± 3.6 |
| 0.125 | + | 21.6 ± 4.2 | 48.1 ± 3.3 | 7.9 ± 2.8 | 22.4 ± 4.8 |
| 0.25 | + | 15.7 ± 1.3 | 54.0 ± 3.4 | 10.7 ± 0.9 | 19.6 ± 3.9 |
| 0.5 | + | 16.0 ± 3.1 | 52.7 ± 2.5 | 11.0 ± 4.2 | 19.7 ± 1.9 |
| 2.0 | + | 10.9 ± 1.6 | 58.9 ± 2.3 | 11.1 ± 1.2 | 18.6 ± 3.3 |

Mean data (± range) derived from two determinations.

studies (data not shown), which included a wild-type p53-expressing human cell line (Smith *et al.*, 1994), confirm that the H69 cell line also shows a defect in arrest at $G_1$/S following acute X-irradiation (S. Soués & P.J. Smith manuscript in preparation). Collectively, the results are consistent with the evasion of the $G_1$/S checkpoint by VP-16-treated H69 cells.

During the incubation period of 24–48 h for a continuous (48 h; condition b in Table I) exposure to doses of <0.25 μM VP-16 there is evidence of cells attempting mitosis, whereas at the 2 μM concentration the majority of cells become trapped in $G_2$. Thus, it appears that at low doses, over the first 48 h, cells are progressing through the cell cycle relatively normally except that there is an increasing probability, with time, of a given cell being trapped in $G_2$. In contrast, at the higher concentration, over the first 24 h period, a large proportion of cells are slowed down or blocked in S-phase with a very high probability that any cell delivered to $G_2$ will remain trapped in that cell cycle compartment.

Cells released from a 24 h exposure to low doses of VP-16 (condition c in Table I) re-enter cycle during the subsequent 24 h incubation in fresh medium. This is in contrast to high-dose VP-16-treated cells, which when released at 24 h continue to accumulate in $G_2$ over the following 24 h period and remain blocked in $G_2$ with very few cells attempting mitosis.

### *S-phase delay analysed by BrdUrd incorporation*

Flow cytometric measurements of BrdUrd incorporation were used to analyse S-phase delay in order to determine the proportion of cells synthesising DNA in treated and untreated cultures. Gates were set around two regions on contour plots of DNA content and BrdUrd incorporation. Region 1 contained cells that did not incorporate BrdUrd, and this region was analysed by the standard cell cycle phase-fitting programme and the calculated $G_1$, 'inactive' S and $G_2$ percentages converted into percentages of total cells within regions. Region 2 was designated 'active S-phase', as this contained cells that incorporated BrdUrd above background levels. Typical results, shown in Table II, indicate that with increasing VP-16 dose after 24 h there is an increase in the percentage of cells engaged in DNA synthesis. The reduced $G_2$ arrest observed upon VP-16 treatment may arise from a direct effect of the thymidine analogue on cell cycle traverse. The contour plots show that the extent of BrdUrd incorporation relative to position in S-phase is similar for control and VP-16-treated cells (data not shown). Parallel studies using thymidine incorporation also show that such DNA synthesis detected in VP-16-treated cells is resistant to the inhibitory effects of acute 1 h high-dose exposures to VP-16 despite continued sensitivity to the DNA–protein cross-linking action of such high doses of drug (data not shown). The results consolidate the stathmokinetic results in that at the 2 μM VP-16 dose level initially delayed cells continue to traverse S-phase after a 24 h drug exposure in an apparently normal manner. Importantly, the percentage of cells in 'inactive' S-phase did not increase above the numbers gated in control cultures in response to VP-16 treatment, suggesting that no cells are actively blocked at 24 h in S-phase for any of the doses studied.

### *Cell cycle delay and recovery in synchronised cultures*

Figure 3a and b shows the effects of VP-16 on the traverse of S-phase as monitored by $G_2$ accumulation. Asynchronous cultures were compared with those released from partial synchrony in early S-phase achieved by aphidicolin treatment. Control cells, released from $G_1$/S synchrony, traverse S-phase during the first 6 h of release, pass as a cohort through $G_2$ and re-enter $G_1$ by 24 h. Following release into 0.25 or 2 μM VP-16 (Figure 3b), cells are delayed in S-phase and accumulate in $G_2$. After a 24 or 30 h VP-16 exposure only cells exposed to the low dose (0.25 μM) can exit $G_2$ and

Figure 3 Effects of VP-16 on S-phase traverse, monitored by $G_2$ accumulation, for asynchronous H69 cells (a) and cells released from synchrony $G_1$/S (b). Mean data derived from three independent experiments (s.e. <10%). Cultures were treated for 6, 24 or 30 h with VP-16 at 0 μM (□), 0.25 μM (▨) or 2 μM (■). Following 24 or 30 h treatments cells were released for the indicated recovery (R) period.

re-enter $G_1$. Synchronisation increases the number of cells experiencing early S-phase during VP-16 exposure and reduces the number of cells capable of recovery from $G_2$ arrest compared with asynchronous cultures. Asynchronous cultures contain fewer cells in early S-phase and show a greater recovery from 0.25 μM VP-16 compared with synchronised cultures. Cells exposed to 2 μM VP-16, under either culture condition remain trapped in $G_2$, indicating the essentially irreversible nature of the arrest for the lowest concentration capable of inducing an overall S-phase delay.

### *DNA damage as a function of cell cycle position*

We have used an adaptation of the 'comet' assay (Singh *et al.*, 1988; Smith & Sykes, 1992) to detect DNA breakage resulting from cleavage of trapped topoisomerase complexes or secondary fragmentation events as monitored by the nucleoid volume measurements. Figure 4 a–c shows the essentially linear relationship between nucleoid volume and DNA content for control cultures and the increase in the proportion of cells with high DNA contents in VP-16-treated cultures. The results reveal heterogeneity in the responses of cells to VP-16, with only a few cells (<15%) showing low levels of damage at 0.25 μM VP-16, whereas the majority of cells (>80%) show substantial damage at 2 μM VP-16. The $G_1$ fraction in cultures exposed to 2 μM VP-16 was too infrequent for detailed analysis. However, some cells with DNA contents approximating to S-phase showed no significant levels of DNA damage and may represent a subpopulation not undergoing either S-phase delay or elevation of damage levels.

Figure 4 Induction of DNA damage in individual H69 cells by 24 h exposure to VP-16 as a function of total cellular DNA content determined by nucleoid volume (halo assay). a, Control cells, line fitted by linear regression ($y = 0.934 + 0.388x$; $R^2 = 0.718$; reproduced in b and c). b and c, 0.25 μM or 2 μM VP-16 × 24 h respectively.

### *VP-16-induced changes in topoisomerase II availability*

*Whole population studies* Immunoblots of whole-cell preparations of H69 cultures exposed to VP-16 for 24 h showed a major immunoreactive band with a mean molecular weight (± s.d. for ten determinations) of 171.3 ± 2.9 kDa corresponding to that expected for the p170 form of human DNA topoisomerase IIα. An increase in the intensity of the p170 band above control was observed for all VP-16-treated samples, and densitometry (Figure 5) was used to quantify the changes with respect to control samples. The p170 band intensity increased with drug dose and exposure period, reaching a maximum of 3.7-fold at doses of 1–2 μM VP-16 with a reduction in band intensity evident at 8 μM VP-16.

*Single-cell analysis and cell cycle distribution* To relate the cell cycle perturbations to the changes in topoisomerase IIα we have utilised a flow cytometric technique (Smith & Makinson, 1989), which provides a simultaneous analysis of DNA content and nuclear DNA topoisomerase II. Initial studies established the cell cycle distribution of topoisomerase II. Bivariate plots of DNA versus topoisomerase II content revealed the expected distribution of the p170 form of DNA topoisomerase II throughout the cell cycle: low levels in $G_1$,

**Figure 5** Densitometric analysis of the p170 topoisomerase II band for Western blots for H69 whole-cell preparations. Cultures were exposed to VP-16 for 24 h (○) or 48 h (●). Data are mean values (± s.d.) derived for four independent blots.

**Figure 6** Histograms of VP-16-induced changes in topoisomerase II, relative to control levels, in cells within gated cell cycle compartments measured 24 h after continuous exposure to 2 μM VP-16 for 24 h. Data are means (± s.e.) of the corrected median values derived from four independent experiments. Median 530 nm RF values were background corrected and expressed as a ratio of the value obtained from unperturbed control cells such that a value of zero indicates no change in topoisomerase II-associated immunofluorescence compared with the control. S phase has been divided into four equal compartments of increasing DNA content.

levels increasing through S-phase, high levels in $G_2$ with a subset in $G_2$ showing the highest levels before recycling into $G_1$. Setting the anti-topoisomerase II antibody staining of $G_1$ cells at unity, signal was increased $1.7 \pm 0.18$-fold and $3.47 \pm 0.61$-fold for S-phase and $G_2$/M cells respectively (± s.d.; three experiments). Extensive redistribution of cells in the cell cycle by exposure to colcemid (see above) for 24 h gave corresponding values of $1.85 \pm 0.28$ and $3.47 \pm 0.19$ for S-phase and $G_2$/M cells respectively, showing that the analysis for immunoreactivity is independent of the proportions of cells within the gated regions. The control data (not shown) demonstrate the progressive increase in nuclear enzyme content through the cell cycle and support the interpretation that the changes seen above by Western blotting represent, at least in part, cell cycle redistribution. DNA content analysis for drug-treated cells (2 μM VP-16 for 24 h) clearly showed the expected VP-16-induced accumulation of cells in $G_2$ and S phase. DNA distributions were divided into six compartments of increasing DNA content representing: $G_1$, early S, early to mid S, mid to late S, late S and $G_2$/M fractions respectively (Figure 6). Median 530 nm fluorescence intensities were calculated for each gated compartment, corrected for background fluorescence and expressed as a value relative to control samples (Figure 6). No increase above control values was observed for cells exposed to 0.25 μM VP-16 (data not shown). At 2 μM VP-16 there is an unscheduled increase in nuclear topoisomerase II content in S-phase and in $G_2$/M.

### Discussion

This study has demonstrated the strict dose dependency of the cell cycle perturbations underlying the cytostatic action of VP-16 on a human SCLC cell line. The disruption of the traverse of S-phase, in particular a delay in early S-phase progression appears to be a key component in the irreversible cytostatic action of VP-16. Continuous exposure to low doses of VP-16 ($\leqslant$0.25 μM) results in significant enrichment of cells within cell cycle compartments which normally express high levels of topoisomerase II. VP-16 induced a cell cycle block in $G_2$ with a significant delay of cells in S-phase being apparent at high VP-16 concentrations. Similar observations have been reported for other human cells including transformed fibroblasts (Smith *et al.*, 1986), lymphoblasts (Kalwinsky *et al.*, 1983) and breast tumour cells (Epstein *et al.*, 1988).

Dysfunction of the p53 proto-oncogene would be expected to contribute to a loss of the ability of cells to arrest at $G_1$/S in response to DNA damage. X-irradiation of the p53 mutant cell line H69 resulted in no detectable arrest of cells at $G_1$/S but progressive accumulation at the $G_2$/M boundary (S. Soués & P.J. Smith, unpublished data). The results obtained with protracted VP-16 exposure also reveal an inability to arrest at $G_1$/S. In viewing the relevance of the concentration dependency of VP-16 effects, it is important to relate the drug doses used in this model study with those found in clinical practice. In a study by Slevin *et al.* (1989*a*,*b*), pharmacokinetic measurements on previously untreated patients with SCLC demonstrated that a 5 day oral regimen could maintain plasma VP-16 levels above 1.7 μM. Thus the present study describes cellular effects at clinically relevant doses of VP-16. The relationship between S-phase delay and subsequent cellular recovery from $G_2$ arrest was studied in synchronised cells exposed to VP-16 upon release from the $G_1$/S transition point. Cells not experiencing a VP-16-induced S-phase delay entered a long-term $G_2$ arrest dependent upon the continued presence of VP-16. Removal of VP-16 resulted in the progression of the majority of these cells through mitosis during the 6–24 h period after drug removal. Cells experiencing a significant VP-16-induced S-phase delay remained in long-term $G_2$ arrest with no evidence of progression from $G_2$.

Continuous exposure to an irreversibly cytostatic concentration of VP-16 also results in an increased immunoreactivity of nuclei to an anti-topoisomerase II antibody. We suggest that two effects can account for this observation. First, the principal effect is an unscheduled increase in topoisomerase II levels. This increase arises from the delay of cells in S-phase during a period in which there is a scheduled increase in topoisomerase IIα. Accordingly, there may be no cellular feedback to link cell cycle progression to topoisomerase levels once a cell is committed to active DNA synthesis. Such a model should be examined with DNA synthesis-inhibiting agents that are not discrete topoisomerase poisons and for the modulation of other cell cycle-regulated proteins. Second, a minor component of the increase may represent enhanced stabilisation of cleavable complexes. In support of this latter possibility is the observation that the sensitive nucleoid expansion method, performed under conditions which cleave DNA at trapped complexes, reveals significant levels of DNA fragmentation in delayed cells. However, the levels of cleavage detected are commensurate with only low levels of complex trapping detectable by the conventional $K^+$SDS precipitation method (P.J. Smith & S.J. Falk, unpublished data).

It does not appear that the fragmentation revealed by nucleoid scanning represents typical apoptosis given the results of the viability measurements and our observation that acridine orange-stained preparations showed that <1% of cells displayed typical apoptotic nuclei. Furthermore, extractions of low molecular weight DNA fractions from VP-16-treated cell populations showed no detectable levels of nucleosome laddering as assessed by conventional agarose gel electrophoresis (P.J. Smith, unpublished data). The lack of early induction of apoptosis is not surprising since it has been reported that SCLC cell lines may differ in their ability to express VP-16-induced apoptosis (Okamoto-Kubo *et al.*, 1994) and the potential requirement of functional p53 for efficient induction (Clarke *et al.*, 1993; Lowe *et al.*, 1993). Additional studies are required to determine whether the damage visualised by nucleoid scanning represents target enzyme trapping or a secondary process of preapoptotic, perhaps DNA domain-limited, fragmentation.

We suggest that the minimal cytotoxic dose threshold for low-dose, protracted VP-16 exposures is defined by the dose intensity required to impose an early S-phase delay rather than effect G2 arrest *per se*. In the *in vivo* situation, the maintenance of a low but bioactive drug concentration would allow the continued recruitment of tumour cells into S-phase delay as they opt to enter the cell cycle since it is unlikely that the $G_1/S$ checkpoint is functional in SCLC and $G_1$ emptying is not affected even by high drug doses. It is evident that SCLC cells can overcome the initial restriction to early S-phase traverse even in the continued presence of VP-16 and cells eventually enter a $G_2$ delayed state with enhanced availability of topoisomerase II.

Clinical studies have supported the concept that prolonged low-dose VP-16 treatment offers the combined benefits of efficacy and low toxicity. On the other hand, it is clear that such treatment will not break new ground in terms of increasing response duration or survival either when used alone (Slevin *et al.*, 1989*a*,*b*) or as part of a conventionally designed regimen (Murphy *et al.*, 1992). However, prolonged schedules of VP-16 treatment may be a foundation for novel combination regimens which capitalise on the consequences of continuous topoisomerase II poisoning such as cell cycle synchronisation or modulation of topoisomerase II levels. This hypothesis was evaluated by exposing H69 cells to VP-16 for 24 h prior to treating washed cultures with selected agents and assaying growth potential using the conventional MTT assay (data not shown). The results indicated that VP-16 pretreatment results in a greater than 2-fold enhancement of growth inhibition potential for cisplatin, Amsacrine (*m*AMSA) and VP-16, while the other agents (camptothecin, mitoxantrone and doxorubicin) gave values of less than 1.3-fold enhancement.

The absence of an effect on sensitivity to the topoisomerase I poison camptothecin is consistent with the non-cell cycle-regulated nature of the target enzyme (Heck *et al.*, 1988). VP-16 pretreatment did not interfere with the cytotoxic potential of the topoisomerase poisons doxorubicin and mitoxantrone, suggesting that the initial capacity to induce topoisomerase II cross-linking not being an important factor in the cytotoxic action of anthracyclines and related drugs (Fox & Smith, 1990; Smith *et al.*, 1990). The >2-fold enhancement of cytotoxicity observed for the topoisomerase II poisons *m*AMSA and VP-16 is itself consistent with the effects of topoisomerase II modulation. The observations of enhanced cisplatin sensitivity is interesting given the capacity of this agent to induce DNA-DNA and DNA-protein cross-linking in what may be topologically compromised DNA molecules in VP-16-pretreated cells.

The implications for cancer chemotherapy are that dynamic changes in cell cycle distribution and target enzyme presentation in tumour cells exposed for protracted periods to low doses of VP-16 may offer novel opportunities for the introduction of other agents in combined regimens. The single-cell analytical approach described here offers a method of monitoring the effects of chronic VP-16 exposure *in vivo* within defined tumour target populations.

### References

CALLAHAN, R. (1992). p53 mutations, another breast cancer prognostic factor. *J. Natl Cancer Inst.*, **84**, 826-827.

CLARK, P.I., JOEL, S.P. & SLEVIN, M.L. (1989). A pharmacokinetic hypothesis for the clinical efficacy of etoposide in small cell lung cancer. *Proc. Am. Soc. Clin. Oncol.*, **8**, 66.

CLARK, P.I., COTTIER, B., JOEL, S.P., THOMPSON, P.I. & SLEVIN, M.L. (1990). Prolonged administration of single-agent oral etoposide in patients with untreated small cell lung cancer. *Proc. Am. Soc. Clin. Oncol.*, **9**, 226.

CLARK, P.I., COTTIER, B., JOEL, S.P. & SLEVIN, M.L. (1991). Two prolonged schedules of single-agent oral etoposide of differing duration and dose in patients with untreated small cell lung cancer. *Proc. Am. Soc. Clin. Oncol.*, **10**, 268.

CLARKE, A.R., PURDIE, C.A., HARRISON, D.J., MORRIS, R.G., BIRD, C.C., HOOPER, M.L. & WYLLIE, A.H. (1993). Thymocyte apoptosis induced by p53-dependent and independent pathways. *Nature*, **362**, 849-852.

DOMBERNOWSKY, P. & NISSEN, N.I. (1973). Schedule dependency of the antileukaemic activity of the podophyllotoxin-derivative VP-16-213 (NSC-141540) in L1210 leukaemia. *Acta Pathol. Microbiol. Scand.*, **81**, 715-724.

EINHORN, L.H., PENNINGTON, K. & McCLEAN, J. (1990). Phase II trial of daily oral VP-16 in refractory small cell lung cancer: a Hoosier Oncology Group study. *Semin. Oncol.*, **17** (Suppl. 2), 32-35.

EPSTEIN, R.J., WATSON, J.V. & SMITH, P.J. (1988). Subpopulation analysis of drug-induced cell-cycle delay in human tumour cells using 90° light scatter. *Cytometry*, **9**, 349-358.

FOX, M.E. & SMITH, P.J. (1990). Long-term inhibition of DNA synthesis and the persistence of trapped topoisomerase II complexes in determining the toxicity of the antitumour DNA intercalators mAMSA and mitoxantrone. *Cancer Res.*, **50**, 5813-5818.

GLISSON, B.S. & ROSS, W.E. (1987). DNA topoisomerase II: a primer on the enzyme and its unusual role as a multidrug target in cancer chemotherapy. *Pharmacol. Ther.*, **32**, 89-106.

HAINSWORTH, J.D., JOHNSON, D.H., FRAZIER, S.R. & GRECO, F.A. (1989). Chronic daily administration of oral etoposide - a phase I trial. *J. Clin. Oncol.*, **7**, 396-401.

HECK, M.M., HITTELMAN, W.N. & EARNSHAW, W.C. (1988). Differential expression of DNA topoisomerases I and II during the eukaryotic cell cycle. *Proc. Natl Acad. Sci. USA*, **85**, 1086-1090.

HOLLSTEIN, M., SIDRANSKY, D., VOGELSTEIN, B. & HARRIS, C.C. (1991). p53 mutations in human cancers. *Science*, **253**, 49-53.

JOHNSON, D.H., GRECO, F.A., STRUPP, J., HANDE, K.R. & HAINSWORTH, J.D. (1990). Prolonged administration of oral etoposide in patients with relapsed or refractory small cell lung cancer: a phase II trial. *J. Clin. Oncol.*, **8**, 1613-1617.

KALWINSKY, D.K., LOOK, A.T., DUCORE, J. & FRIDLAND, A. (1983). Effects of Epipodophyllotoxin VP-16-213 on cell cycle traverse DNA synthesis and DNA strand size in cultures of human leukemic lymphoblasts. *Cancer Res.*, **43**, 1592-1597.

KARN, J., WATSON, J.V., LOWE, A.D., GREEN, S.M. & VEDECKIS, W. (1989). Regulation of cell cycle duration by c-*myc* levels. *Oncogene*, **4**, 773-787.

KASTAN, M.B., ZHAN, Q., EL-DEIRY, W.S., CARRIER, F., JACKS, T., WALSH, W.V., PLUNKETT, B.S., VOGELSTEIN, B. & FORNACE, Jr. A.J. (1992). A mammalian cell cycle checkpoint pathway utilizing p53 and GADD45 is defective in ataxia-telangiectasia. *Cell*, **71**, 587-597.

LANE, D.P. (1992). p53, guardian of the genome. *Nature*, **358**, 15-16.

LEVINE, A.J., MOMAND, J. & FINLAY, C.A. (1991). The p53 tumour suppressor gene. *Nature*, **351**, 453-456.

LIU, L.F. (1989). DNA topoisomerase poisons as antitumour drugs. *Annu. Rev. Biochem.*, **58**, 351-375.

LIVINGSTONE, L.R., WHITE, A., SPREOUSE, J., LIVANOS, E., JACKS, T. & TLSTY, T.D. (1992). Altered cell cycle arrest and gene amplification potential accompany loss of wild-type p53. *Cell*, **70**, 923-935.

LOCK, R.B. & ROSS, W.E. (1990). Possible role for p34cdc2 kinase in etoposide-induced cell death of Chinese hamster ovary cells. *Cancer Res.*, **50**, 3767-3771.

LOWE, S.W., RULEY, H.E., JACKS, T. & HOUSMAN, D.E. (1993). p53-dependent apoptosis modulates the cytotoxicity of anticancer agents. *Cell*, **74**, 957-967.

MILLER, A.A., STEWART, C.F. & TOLLEY, E.A. (1990). Clinical pharmacodynamics of continuous-infusion etoposide. *Cancer Chemother. Pharmacol.*, **25**, 361-366.

MINFORD, J., POMMIER, Y., FILIPSKI, J., KOHN, K.W., KERRIGAN, D., MATTERN, M., MICHAELS, S., SCHWARTZ, R. & ZWELLING, L.A. (1986). Isolation of intercalator-dependent protein-linked DNA strand cleavage activity from cell nuclei and identification as topoisomerase II. *Biochemistry*, **25**, 9-16.

MURPHY, P.B., HAINSWORTH, J.D., GRECO, F.A., HANDE, K.R., DEVORE, R.F. & JOHNSON, D.H. (1992). A phase II trial of cisplatin and prolonged administration of oral etoposide in extensive stage small cell lung cancer. *Cancer*, **69**, 370-375.

OKAMOTO-KUBO, S., NISHIO, K., HEIKE, Y., YOSHIDA, M., OHMORI, T. & SAIJO, NAGAHIRO, S. (1994). Apoptosis induced by etoposide in small-cell lung cancer cell lines. *Cancer Chemother. Pharmacol.*, **33**, 385-390.

SINGH, N.P., MCCOY, M.T., TICE, R.R. & SCHNEIDER, E.L. (1988). A simple technique for quantitation of low levels of DNA damage in individual cells. *Exp. Cell Res.*, **175**, 184-191.

SLEVIN, M.L., CLARK, P.I., JOEL, S.P., MALIK, S., OSBORNE, R.J., GREGORY, W.M., LOWE, D.G., REZNEK, R.H. & WRIGLEY, P.F.M. (1989*a*). A randomised trial to evaluate the effect of schedule on the activity of etoposide in small cell lung cancer. *J. Clin. Oncol.*, **7**, 1333-1340.

SLEVIN, M.L., CLARK, P.I., JOEL, S.P., MALIK, S., OSBORNE, R.J., GREGORY, W.M., LOWE, D.G., REZNEK, R.H. & WRIGLEY, P.F.M. (1989*b*). A randomised trial to examine the effect of more extended scheduling of etoposide administration in small cell lung cancer. *Proc. Am. Soc. Clin. Oncol.*, **8**, 236.

SMITH, P.J. & MAKINSON, T.A. (1989). Cellular consequences of overproduction of DNA topoisomerase II in an ataxia-telangiectasia cell line. *Cancer Res.*, **49**, 1118-1124.

SMITH, P.J. & SYKES, H. (1992). Simultaneous measurement of cell cycle phase position and ionizing radiation-induced DNA strand breakage in single humen tumour cells using laser scanning confocal imaging. *Int. J. Radiat. Biol.*, **61**, 553-560.

SMITH, P.J., ANDERSON, C.O. & WATSON, J.V. (1986). Predominant role for DNA damage in etoposide-induced cytotoxicity and cell cycle perturbation in human SV-40-transformed fibroblasts. *Cancer Res.*, **46**, 5641-5645.

SMITH, P.J., MORGAN, S.A., FOX, M.E. & WATSON, J.V. (1990). Mitoxantrone-DNA binding and the induction of topoisomerase II associated DNA damage in multidrug-resistant small cell lung cancer cells. *Biochem. Pharmacol.*, **40**, 2069-2078.

SMITH, P.J., SOUES, S., FALK, S.J. & HILL, B.T. (1994). G1/S checkpoint evasion and resistance to a topoisomerase II poison (doxorubicin) in human breast tumor cell lines. *Proc. Am. Assoc. Cancer Res.*, **35**, A147.

TAKAHASHI, T., NAU, M.M., CHIBA, I., BIRRER, M.J., ROSENBERG, R.K., VINOCOUR, M., LEVITT, M., PASS, H., GAZDAR, A.F. & MINNA, J.D. (1989). p53: a frequent target for genetic abnormalities in lung cancer. *Science*, **246**, 491-494.

TAKAHASHI, T., SUZUKI, H., HIDA, T., SEKIDO, Y., ARIYOSHI, Y. & UEDA, R. (1991). The p53 gene is very frequently mutated in small-cell lung cancer with a distinct nucleotide substitution pattern. *Oncogene*, **6**, 1775-1778.

WANG, J.C. (1985). DNA topoisomerases. *Annu. Rev. Biochem.*, **54**, 665-697.

WATSON, J.W., CHAMBERS, S.H. & SMITH, P.J. (1987). A pragmatic approach to analysis of DNA histograms with a definable G1 peak. *Cytometry*, **8**, 1-8.

WOLFE, S.N., GROSH, W.W., PRATER, K. & HANDE, K.R. (1987). *In vitro* pharmacodynamic evaluation of VP-16-213: implications for chemotherapy. *Cancer Chemother. Pharmacol.*, **19**, 246-249.